# Perspectives from Systems-Level Key Informants on Optimizing Opioid Use Disorder Treatment for Adolescents and Young Adults

**DOI:** 10.3390/children12070876

**Published:** 2025-07-02

**Authors:** Jasper Yeh, Crosby Modrowski, Isabel Aguirre, Samantha Portis, Robert Miranda, Melissa Pielech

**Affiliations:** 1 Center for Alcohol and Addiction Studies, Brown University School of Public Health, 121 South Main Street, Providence, RI 02903, USA; jasper_yeh@brown.edu (J.Y.); isabel_aguirre@alumni.brown.edu (I.A.); samanthaportis@missouri.edu (S.P.); robert_miranda_jr@brown.edu (R.M.J.); 2 Department of Psychiatry and Human Behavior, The Warren Alpert Medical School of Brown University, 222 Richmond Street, Providence, RI 02903, USA; crosby_modrowski@brown.edu; 3 Bradley Hasbro Children’s Research Center, 1 Hoppin Street, Providence, RI 02903, USA

**Keywords:** opioid, opioid use disorder, opioid use disorder treatment, youth, adolescent, substance use disorder, qualitative

## Abstract

**Background/Objectives:** Rates of receiving opioid use disorder (OUD) treatment among adolescents and young adults (AYA) aged 16–25 are low. The current study qualitatively analyzed informants’ perspectives regarding the availability of, developmental considerations relevant to, and barriers associated with OUD treatment for AYA. **Methods**: Thirty key informants involved with OUD treatment in the northeastern United States completed individual, semi-structured interviews, including treatment providers (N = 11) and clinic leaders in programs that provide medication and psychosocial treatments for AYA with OUD (N = 10), as well as opioid-related policymakers (N = 6) and patient advocates (N = 3). Interviews were transcribed and independently double coded. Template-style thematic analysis methods were used and revealed seven themes. **Results**: The first theme highlighted limited treatment program availability for adolescents (aged < 18 years) with OUD. Four themes related to developmentally optimizing OUD treatment for AYA, describing the importance of caregiver involvement, AYA peer connections, wraparound services, and early intervention. Two themes described barriers to AYA OUD treatment, including stigma and knowledge gaps about medications for OUD as well as deficits in AYA’s access to basic resources (e.g., housing, food security) that prohibit effective participation in treatment. **Conclusions**: Results highlight concerns from systems-level key informants regarding gaps in OUD treatment options for youth under the age of 18 and a high need for OUD treatment that is developmentally tailored to AYA. Findings point toward potential modifications and additions to existing adult treatment programs to make OUD treatment more accessible, relevant, and engaging for AYA.

## 1. Introduction

Adolescents and young adults (AYA) contribute significantly to, and are heavily impacted by, the ongoing opioid crisis in the United States. It is estimated that 406,000 adolescents aged 12–17 years, along with 1.1 million young adults aged 18–25 years, report past-year misuse of prescription opioids (i.e., nonprescription use or use in a manner different than indicated by a healthcare professional), while 265,000 adolescents and 424,000 young adults meet criteria for opioid use disorder (OUD) in the U.S. in the past year [1]. Furthermore, between 1999 and 2018, opioid overdose deaths among AYA increased by 384% [2], and since 2020, adolescents have experienced a greater relative increase in drug overdose deaths than the overall population, which is largely attributed to the increasing prevalence of fentanyl [3]. Apart from high mortality rates, opioid use among AYA is also linked to a host of co-occurring psychiatric disorders, increased rates of school dropout, involvement with the criminal justice system, infectious disease associated with injection drug use, and progression to OUD, underscoring the importance of early intervention [4].

**Availability of OUD treatment for AYA:** Evidence-based treatments for AYA with OUD are crucial for decreasing opioid-related morbidity and mortality. Although evidence-based substance use disorder treatments exist for AYA, only 3.5% of adolescents and 4.1% of young adults in the U.S. with past-year substance use disorder, including OUD, received treatment [5]. Among AYA who experienced non-fatal opioid overdoses, 68.9% did not receive any opioid-related treatment services within thirty days of overdose, further highlighting the abysmal rates of treatment access in this population [6].

**Developmental considerations for OUD treatment for AYA:** National organizations across the U.S. recommend that effective OUD treatment for AYA include both medication and psychosocial treatments [7,8]. However, AYA’s access to medications for OUD (MOUD), such as methadone and buprenorphine, and effective psychosocial treatment is limited, especially for adolescents. According to U.S. federal regulations, methadone is approved for those at least 18 years old, with underage prescribing requiring documentation of two previously failed non-pharmacological treatments, while buprenorphine is approved for those at least 16 years old [9]. However, adolescents who are eligible for MOUD are much less likely to receive it than adults, with only 2.4% and 0.4% of adolescents in treatment for heroin and prescription opioid use, respectively, receiving MOUD [10]. Furthermore, there is a dearth of AYA-specific treatment programs available, and many programs do not accept those younger than 18 years old [11]. Admitting AYA to adult treatment programs risks overlooking the unique developmental characteristics of this age group, such as higher risk of co-occurring mental health diagnoses and infectious disease, as well as complex developmentally centered needs surrounding family dynamics, education, employment, and legal issues [11]. It is perhaps not surprising, then, that AYA encounter unique barriers to accessing substance use treatment, such as limited family engagement, lower motivation for treatment, and difficulty in adjusting to residential treatment settings [12].

**Barriers to providing OUD treatment to AYA:** Expanding access to evidence-based substance use treatments for AYA requires an understanding of the existing barriers and facilitators to care. Indeed, specialty care for OUD presents somewhat unique challenges for many AYA, but little is known regarding what clinics perceive as barriers to addressing the needs of AYA with OUD, and OUD treatment providers may be well positioned to help clarify these barriers and devise ways to overcome them. Research has not assessed barriers to accessing OUD treatment for AYA from the perspective of those involved in the provision of OUD treatment, and doing so could yield highly relevant practical information for current and future treatment providers.

Therefore, the current analysis sought to fill this critical gap and qualitatively analyze interview content from key informants, namely clinicians, clinic administrators, patient advocates, and policymakers, who are knowledgeable about the OUD treatment system in the northeastern US states of Rhode Island and Massachusetts. While it is important to acknowledge that there is no replacement for the inclusion of AYA and their parents in studying AYA OUD treatment, the current study represents a formative study to collect systems-level feedback that is intended as an initial step to inform program development before future study efforts that will directly involve AYA and their family.

Template-style thematic analysis methods were utilized to summarize key informant perspectives on: (1) availability of OUD treatment for AYA, (2) developmental considerations for optimizing OUD treatment for AYA, and (3) barriers to providing OUD treatment to AYA. Results of this study can be used to inform developmentally tailored programming for treating AYA with OUD to ensure that these services are accessible and catered to the specific needs of AYA.

## 2. Materials and Methods

Data for this analysis were sourced from a qualitative study with key informants from community opioid and other substance use treatment programs in Rhode Island and Massachusetts that serve adolescents, young adults, or both. The primary aim of the parent qualitative study was to identify systems-level barriers and facilitators to family involvement in youth OUD treatment based on the Consolidated Framework for Implementation Research in order to inform the development of a survey to quantify these barriers [13]. As part of the qualitative interviews, key informants were also asked to discuss the availability of OUD treatment for youth, developmental considerations for providing youth OUD treatment, and barriers to offering OUD treatment to youth. The current study focuses on interview content centered on these topics. This qualitative data has not been previously analyzed or published.

Participant recruitment occurred from December 2019 to April 2020 via direct outreach to treatment clinics, relevant policymakers, and advocates, along with snowball methods until reaching data saturation, in which further interviews began to yield redundant themes [14,15]. The Brown University Institutional Review Board reviewed the study protocol and procedures and deemed that study participants were key informants rather than human subjects; thus, IRB approval was not needed.

Sampling for the study was purposive. Eligible participants were (1) frontline treatment providers at programs that offer MOUD, (2) leaders of treatment programs that offer MOUD, (3) patient advocates, or (4) relevant policymakers. Eligible frontline providers had to carry an active caseload and have experience providing psychosocial support to patients receiving MOUD. Eligible leaders were part of the leadership staff for an OUD treatment program and supervised frontline providers. Eligible patient advocates either had lived experience with OUD or worked with youth with OUD, along with their families, for at least six months. Eligible policymakers or community leaders actively held a state-level position, with responsibility for opioid-related programming, for at least six months. Demographic and professional characteristics of participants were collected and summarized using descriptive statistics.

Semi-structured interviews lasting approximately 45 min were administered via Zoom or telephone, based on participant preference. Interviews were primarily administered by the senior author, a doctoral-level researcher and licensed clinical psychologist with expertise in youth substance use and family-based treatment approaches, or a trained study staff member with close supervision by the senior author. Interview questions relevant to this study focused on three areas: availability of OUD treatment for AYA, developmental considerations for optimizing OUD treatment for AYA, and barriers to providing OUD treatment to AYA. Table 1 lists the interview questions from this study that are relevant to this analysis. All participants received a $50 gift card for their time.

Template-style thematic analysis approaches were used for qualitative analysis [16] due to their balance of structure and flexibility for coding textual data such that an initial coding template could be developed based on the semi-structured interview guide (deductive) and iteratively modified to reflect emergent, salient themes in the interview data (inductive).

All interviews were audio recorded and transcribed verbatim. Interview transcripts were uploaded to NVivo 14, a qualitative data analysis software, for coding. An initial coding template was drafted based on the semi-structured interview questions. All coders were trained to use the codebook, collectively coded an initial set of interviews, and achieved consensus on how to use each code. The codebook was edited and refined until all coders agreed that the code names and definitions could adequately represent all interview content. Subsequent interview transcripts were each independently coded by two coders who then met to resolve discrepancies as well as observe and reflect on bias until 100% agreement in coding was reached.

Content within each code was reviewed iteratively to look for emerging themes, in line with the template-style thematic analysis methods. Codes examined in the current analysis are presented in Table 1. All generated themes were then reviewed together, with themes across different codes compared, consolidated, and modified until they adequately represented all thematic content present in the codes being studied. Representative quotes that best illustrate each theme were then compiled.

## 3. Results

Thirty participants were interviewed, including 11 treatment providers, 10 clinic leaders, 3 patient advocates, and 6 policymakers. Most (80.0%) participants identified as female, and 93.3% identified as White. Nearly two-thirds (60.1%) of participants had over two years of experience in their current agency, while 26.7% had over a decade of experience in their current agency. All participants had a bachelor’s degree or higher, with 67.7% having a Master’s, PhD, or MD degree. Table 2 describes the demographics of key informants involved in this study.

**Figure 1:**
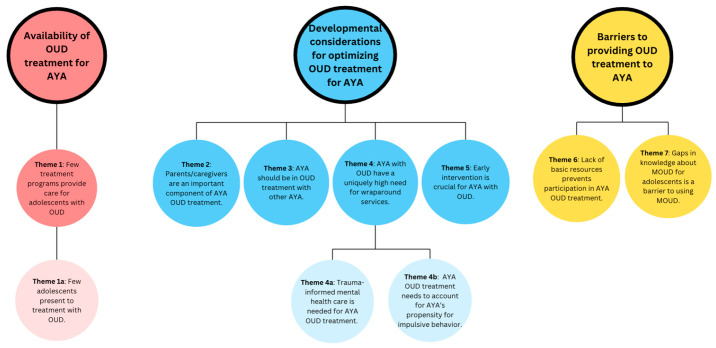
A summary of all seven themes, along with three subthemes, organized into three primary areas of interest.

Through template-style thematic analysis, seven themes emerged, along with three subthemes. These themes are organized based on the three main areas of interest of our study. The first theme describes the availability of OUD treatment for AYA. The second, third, fourth, and fifth themes discuss developmental considerations for optimizing OUD treatment for AYA. The sixth and seventh themes describe barriers to providing OUD treatment for AYA. Figure 1 provides a summary of all themes organized into these three main areas of interest.

### 3.1. Availability of OUD Treatment for AYA

The first theme was generated from asking participants their perspectives on the current local availability of OUD treatment for AYA.

#### 3.1.1. Theme 1: Few Treatment Programs Provide Care for Adolescents with OUD, Despite a Need for OUD Treatment Among This Age Group

Participants recognized that there is a crucial need for OUD treatment for the AYA population and acknowledged that there are not enough treatment resources available for those younger than 18 years because many programs do not accept adolescents. As one participant said, “the need for all adolescent substance use…is large and the resources are small…then if you’re talking about the resources that are providing a really solid evidence-based [care]…it then whittles down even further” (clinic leader). Many participants said there is very limited higher-level OUD care for adolescents, including a lack of detoxification programs, residential treatment centers, and intensive outpatient programs that can accommodate adolescents.

##### Theme 1a: Few Adolescents Present to Treatment with OUD, Making It Difficult for Treatment Programs to Sustain Adolescent-Specific Programming

One participant, whose program tried to create an adolescent-specific program, said “it wasn’t feasible to keep it operational when our census was zero or one for weeks on end” (clinic leader). As a result, participants shared that it can be difficult to advocate for adolescent OUD treatment operations. Similarly, participants shared that prioritizing provider training for the adolescent population was hard to justify due to the low numbers of adolescent patients.

Notably, many participants attributed the lack of adolescents in OUD treatment to difficulties with treatment access rather than a lack of treatment need. Participants gave varying reasons for why they believed so few adolescents present to treatment. Some cited “missed opportunities” to identify these adolescents at emergency departments due to adolescents not being “forthcoming around their actual use patterns” (clinic leader) as well as not experiencing overdoses to the point of presenting at emergency rooms. One participant said, “adolescents, either through luck, better brain power, resilience, I’m not sure what, if they’re using it, they’re not using it to the point of getting to that level of emergency services that they get identified” (policy maker). Another participant added that adolescents often do not know how opioids are defined and thus fail to report their use as such during screening, which negatively impacts referrals to appropriate treatment.

### 3.2. Developmental Considerations for Optimizing OUD Treatment for AYA

Participants were asked about the unique needs of AYA served by their organization, which resulted in participants providing numerous suggestions and considerations for creating developmentally tailored OUD treatment for AYA. Comments about these age-specific considerations are organized into four themes presented below.

#### 3.2.1. Theme 2: Parents/Caregivers Are an Important Component of AYA OUD Treatment Because They Are a Crucial Part of Youth’s Environment. Thus, Psychoeducation and Skills-Building for Parents Would Be Useful for Helping Them to Support Their Youth

Participants repeatedly commented on the importance of including parents/caregivers (herein referred to as “parents”) in the treatment of AYA with OUD. As individuals who are not yet fully independent and are likely relying on parents for housing and financial support, it is critical to understand the youth’s existing support system and living environment. As one participant said: “You need that family context to generalize things and to actually work with the system to make long-standing changes instead of putting a bandaid on a bullet hole with teaching just the kid” (treatment provider).

Many participants stated the importance of providing psychoeducation for parents too, especially in terms of working on building healthy relationships with their children. A few participants spoke of the importance of helping parents navigate, allowing autonomy for their youth as they transition toward independence. One participant said:

“The perception on the part of the caregiver is, ‘well, when you were 10, I could just walk in your room and do whatever, and you also haven’t been behaving yourself, so you’re still in my house, and I can do whatever.’ There’s a huge developmental need…around figuring out how to have privacy and how to earn privacy and how to earn freedom in ways that make sense”.

(clinic leader)

Another participant said that because youth are beginning to attain independence, their parents often do not know how to implement “appropriate or consistent interventions or consequences for kids’ behavior” (treatment provider).

Participants also suggested that parents would benefit from psychoeducation to help them understand AYA substance use and the OUD recovery process. For example, one participant suggested creating a drop-in center where parents can call and ask any questions about their youth’s mental health and substance use. Another participant suggested an introductory group session for parents where they can learn more about substance use and acquire related skills to support their youth.

#### 3.2.2. Theme 3: AYA Should Be in Treatment with Other AYA to Increase Relatability, Establish Peer Recovery Connections, and Share Treatment Experiences That Are Unique to the AYA Population

As one participant said:

“I think having a group that’s specifically tailored to young adults could be nice to talk about some of the unique challenges that present themselves with this population or just feeling like there’s an opportunity to connect with your peers who are going through shared or similar experiences”.

(clinic leader)

Participants said that it is often difficult for AYA to relate to older adults, with one participant saying that if youth “go to AA and NA, they’re gonna see a bunch of 50-year-old guys, and it doesn’t feel like their recovery” (clinic leader). Many participants also emphasized that it may be uncomfortable for AYA to be in the same treatment groups as older adults who have struggled with addiction for much longer. One participant said of AYA:

“As they’ve been exposed to older individuals who’ve been struggling with addiction for a long time…I think it was really kind of terrifying and scared them in a way that wasn’t productive, or they couldn’t relate to that person. They felt that it was just a really different thing than what they were experiencing”.

(clinic leader)

Some participants even remarked that mixing youth with older adults in the same groups can lead to unhealthy outcomes, and can cause youth to be “exposed to things that they shouldn’t be at that young of an age.” One participant mentioned “contagion effect,” saying:

“You have kids that are developmentally impulsive, they hear about somebody in their mid-20’s, it looks pretty cool and they’re doing something novel and they say, ‘well, I’m gonna try that,’ even though in the whatever meeting or group they might have been in, it was meant to be ‘don’t try it”.

(policy maker)

#### 3.2.3. Theme 4: Along with Treatment Focused on OUD, Youth Have a Uniquely High Need for Wraparound Services, Including Mental Healthcare, Development of Basic Life Skills, and Vocational/Educational Assistance

As summarized by one participant:

“I really think that [AYA OUD treatment], if it’s properly done, it needs to be an interdisciplinary team of providers, counselors, social workers, case managers, to make sure that that one patient, one person, getting cared for in a holistic manner on a whole bunch of different levels and dimensions”.

(clinic leader)

Participants commonly spoke of the importance of providing mental healthcare and highlighted the need to help their young clients build psychosocial skills in navigating complex emotions, relationships, and social situations. One participant said:

“When [youth with OUD] stop using, you know, a lot of times the use covered up or masked a lot of their feelings and emotions. And so then they have this flood of kind of feelings and emotions that they’ve never had to cope with before, and suddenly they’re like having to figure out what to do with that”.

(Participant 4, treatment provider)

While mental health treatment was identified as integral to AYA OUD treatment, many participants pointed out that there is a lack of training in addressing co-occurring mental health diagnoses and substance use disorder, creating disjointed care. As one participant said: “People are scared to work with substance use disorders. It’s always ironic to me because it’s like, ‘but you probably already are.’ It just goes under diagnosed and then that need is not met” (clinic leader).

Additionally, participants stated the need for helping AYA in building basic life skills, such as financial management, cooking, and healthy eating. Participants also frequently cited the need for educational and vocational support for AYA undergoing OUD treatment, including offering resources for getting a GED, helping youth enroll in vocational schools, and helping youth access employment opportunities. One participant explained: “Before they have to worry about a bad work history, let’s help set them up to develop a good work history, get them access to jobs” (clinic leader). Participants also suggested that it is important to involve schools in supporting students’ recovery processes, such as by having substance use counselors in schools or by educating school staff on how to best accommodate students with substance use disorders to ensure the continuation of their education throughout OUD treatment.

##### Theme 4a: There Is a High Need to Integrate Trauma-Informed Mental Health Care into OUD Treatment Due to the High Prevalence of Adverse Childhood Experiences (ACEs) Among the AYA OUD Population

One participant said:

“They have so much trauma going on. They need a quick fix to get over depression, anger, anxiety, trauma…or they’ve seen horrible things happen”.

(policymaker)

The participant added that treating these youth means “having them trust somebody and being accepted for who they are even if they do bad things, and even if they do use drugs or say bad things.” Another participant, in describing the importance of addressing trauma at a young age, said, “the earlier you could deal with trauma, sexual abuse, physical abuse, the trauma that’s evolved in a family, the more resilient that adolescents could be” (policymaker).

##### Theme 4b: Developmentally Tailored OUD Treatment Needs to Account for Biopsychosocial Factors That Increase AYA’s Propensity for Impulsive Behavior

Many participants discussed that for AYA, “their prefrontal cortex is not developed yet,” leading them to engage in more risk-taking behavior and make shortsighted decisions. One participant said that “if we wanna encourage impulse control around substance use, that’s potentially even harder for someone whose brain is actually not quite finalized versus a person who’s an adult who’s older” (clinic leader). Another participant said that many youth “don’t think of that five steps down the road something could happen,” but that they are “looking for today’s feel good thing ‘cuz they don’t like what’s going on at home or at school” (policy maker). Participants therefore noted that it is important to meet teens where they are at, considering what they are able, and not able, to do as they engage in treatment. As one participant explained: “They’re really complex kids…you have to manage your expectations as to what is realistic for someone with an adolescent brain to be able to control” (treatment provider).

#### 3.2.4. Theme 5: Early Intervention Is Crucial for AYA with OUD to Prevent Progression to More Severe OUD

Participants spoke of the importance of intervening in AYA OUD as early as possible due to the opportunity to prevent more severe OUD. Unlike older adults, AYA are still developing, which creates a favorable yet critical context for intervention. As one participant said: “Our perspective is catch ‘em early. The earlier you catch ‘em, hopefully, the better probability you have of getting them where they’re gonna be that less addicted as young adults or adults” (policy maker). Another participant said:

“You have a certain trajectory in your life. The more quickly you address deviance from that trajectory the better…If you’re addressing either the pre-opioid uses or the early opioid uses, the chances of impacting the trajectory of their life and more effectively getting back on is gonna be much more effective”.

(policy maker)

### 3.3. Barriers to Providing OUD Treatment for AYA

Participants were asked their perspectives on the barriers that exist to providing OUD treatment to AYA. Comments about these perceived barriers are organized into two themes presented below.

#### 3.3.1. Theme 6: AYA Often Struggle with a Lack of Basic Resources That Prevent Participation in OUD Treatment

Participants mentioned that financial barriers propelled the need for some AYA to work to provide for their families, leaving less time for consistently attending treatment. Housing issues and food security were also brought up as major issues for AYA that prevent effective participation in OUD treatment. As one participant stated: “How do you discuss substances…when you have a client in front of you with zero support, no health insurance, no primary, and doesn’t have food stamps or a roof over their head” (treatment provider). Participants also cited inaccessibility of transportation as a barrier to participating in OUD treatment. One participant explained,

“If it’s working parents, it’s really difficult to get a kid from school to the program on time and then for the parents to make the time, especially if they are financially strained in any way to make the time to participate in family meetings let alone set up that transportation”.

(treatment provider)

With many AYA being newly independent, they frequently do not have many connections with other people that will allow them to access needed resources. One participant said: “One of the biggest issues when they get out of recovery, is all of a sudden they have no friends. They have nowhere to live probably. They have no job, no money, nothing to do” (patient advocate). Another participant added that youth in treatment “aren’t as established as maybe another client, and yet they’re old enough to no longer have maybe a parental influence around,” (treatment provider), which means they have very little network to reach out to for resources.

#### 3.3.2. Theme 7: Stigma and Gaps in Knowledge from Patients, Family Members, and Providers About MOUD for Adolescents Is a Barrier to Using MOUD for This Population

Participants stated that both AYA patients and their family members often wanted the patient to discontinue MOUD as soon as possible. Some participants cited stigma as a reason for wanting to discontinue MOUD, with patients and families fearing developing a dependency on these medications. One participant said: “Even if the buprenorphine is working really well for patients and supporting their recovery, there’s a desire to get off it as quickly as possible and not develop a dependency on it” (clinic leader). Another participant, speaking of the stigma associated with MOUD from the perspective of providers, said that “people are petrified of putting young people on these meds” (patient advocate). Participants also remarked that people often did not understand that MOUD is best used when augmented by behavioral treatment methods. One participant explained that many clients just want to “come get my dose” and then leave, but “they don’t realize they gotta do group therapy, they gotta do individual counseling” (clinic leader).

## 4. Discussion

In this study, key informants who are knowledgeable of the OUD treatment system in the northeastern US states of Rhode Island and Massachusetts participated in semi-structured qualitative interviews focused on the treatment needs of AYA with OUD. Seven themes emerged, along with three subthemes, that describe the availability of OUD treatment for AYA, developmental considerations for optimizing OUD treatment for AYA, and barriers to providing AYA OUD treatment.

Concerns about the availability of OUD treatment for adolescents were particularly salient due to the lack of programs that will accept those under 18 years old, resulting in a major treatment gap for adolescents with OUD. Indeed, existing data indicate a large discrepancy between the number of adolescents who need OUD treatment and the number of adolescents who ultimately access OUD treatment [1,15]. Participants often attributed the lack of adolescent OUD treatment options to the small number of adolescents presenting for OUD treatment. While there are fewer adolescents with OUD as compared to adults [1], there is also a much smaller proportion of AYA with OUD who present to treatment compared to adults [17]. Given the imbalance in supply versus demand, participants reflected on challenges that their programs face in attempting to sustain adolescent-focused programming. This circular challenge also highlights the importance of improved screening and referral to treatment at both primary and acute care settings for AYA, along with the need to better engage youth in treatment. The American Academy of Pediatrics recommends the use of screening, brief intervention, and referral to treatment in addressing youth substance use [18]. However, research suggests that fewer than half of pediatricians use a validated screening tool to identify substance use among AYA [19]. Participants discussed that early identification of opioid use among AYA is key for early intervention, and thus crucial to preventing progression to OUD. Future efforts are needed to educate providers on screening for OUD among AYA, and improvements in screening strategies are necessary in acute care settings.

Participants also often spoke of the barriers against offering MOUD to those younger than 18. Currently, the gold standard for evidence-based OUD treatment for AYA includes the use of pharmacological treatment, namely buprenorphine, combined with psychosocial treatments [20]. While there is extensive evidence that MOUD treatment for adults reduces opioid-related morbidity and mortality, much less research has been conducted for MOUD with adolescents [18,19]. However, existing literature suggests that MOUD is effective in reducing opioid use among older adolescents and does not point to any age-specific safety concerns for using buprenorphine with older youth [8,20]. The use of MOUD for AYA is also affirmed by multiple national organizations in the U.S. [7,8,21]. Still, adolescent access to MOUD remains low, with one study showing that only 24.4% of facilities offering treatment to adolescents prescribed buprenorphine and another study estimating that only approximately 0.4% of adolescents in treatment for prescription opioid use received MOUD [10,22].

Even with the elimination of the DATA-Waiver Program in 2023, which had previously required clinicians to undergo an additional multi-hour training to be authorized to prescribe buprenorphine for OUD treatment, clinicians are still often hesitant to prescribe buprenorphine to adolescents, citing insufficient training and experience prescribing to this population, a dearth of psychosocial support for adolescents, and lack of access to specialists in addiction medicine and psychiatry [23,24]. Additionally, state policies regarding buprenorphine prescription vary across the U.S., with many states continuing to enact restrictive regulations on buprenorphine prescription despite the federal elimination of the X-Waiver [25]. There is also intense stigma against MOUD coming from both AYA and their parents due to the risk of dependency on these medications, a perspective that is represented among study participants and documented in existing qualitative literature [23,24,26]. Efforts are needed to greatly increase access to MOUD for adolescents, which can be performed through expanding the evidence base of using MOUD for adolescents, training providers, especially pediatricians, on offering MOUD, and providing youth and their families with education about the risks and benefits of MOUD so they can make informed choices.

Along with MOUD, psychosocial treatments are also an integral part of OUD treatment, especially for AYA. Indeed, SAMHSA recommends that buprenorphine should be used as “part of a comprehensive management program that includes psychosocial support” [27]. A robust evidence base shows that family-based treatment (FBT) is one of the most effective forms of psychosocial treatment for addressing substance use disorders among AYA and should be used as a first-line therapy for this population [25,28,29]. Consistent with empirical evidence, participants frequently discussed the importance of including family members in AYA OUD treatment. Family involvement is critical due to the family often lying at the center of AYAs’ social systems and the foundational role that family plays in AYAs’ environment throughout development. FBT approaches can come in many forms, but often consist of including family members in treatment sessions, helping to build healthy relationships between parents and AYA clients, and skills-building for parents to support their children during treatment processes [30].

Despite an abundance of evidence showing the effectiveness of FBT approaches, barriers exist that prevent family participation. Many families face transportation issues and work conflicts, and some struggle with substance use themselves [12]. Furthermore, in follow-up work to the parent qualitative study, providers from OUD treatment programs reported a wide range of barriers, including a lack of staff trained in FBT, insufficient funding to expand services to families, not enough time in staff’s schedules, and insurance reimbursement issues [13]. To minimize barriers associated with implementing FBT approaches, Hogue et al. (2017) [31] distilled manualized FBT models into four core components that could be delivered in a more flexible, scalable, and sustainable manner. Indeed, participants in the current study rarely cited specific FBT models, but rather encouraged more opportunities for family members to become involved in different ways during AYA OUD treatment. More evidence-based opportunities to include family members in treatment should therefore be offered so that parents can be involved in ways that are adapted to the needs of each client.

Additionally, participants consistently cited the need for wraparound services throughout AYA OUD treatment, including mental health treatment, basic resources, life skills, as well as educational and vocational assistance. This echoes the National Institute of Drug Abuse (NIDA) principles of effective treatment, which state that psychological, social, legal, vocational, and other medical needs must be addressed during substance use disorder treatment [32]. In fact, providing wraparound services to clients with substance use disorders has been shown to improve treatment retention and treatment outcomes [33,34]. Although the majority of substance use disorder treatment centers provide at least some wraparound services, the average program offers less than half of the wraparound services recommended by NIDA [35]. Importantly, AYA with OUD have particularly high rates of comorbid psychiatric disorders and more frequently experience issues with emotional regulation, impulsivity, and executive control [4]. Participants also discussed the need for addressing the trauma that many AYA with OUD have, which accurately reflects current data that suggests a higher prevalence of ACEs among people with substance use disorders, as well as a positive association between ACEs and subsequent substance use [36]. Together, these points highlight the need for treating concurrent mental health issues during OUD treatment. Furthermore, AYA are often still embedded in the education system or have only recently moved beyond schooling. Therefore, educational and vocational support are especially important in helping AYA to complete an educational degree or develop a favorable work history to increase future career opportunities. Adolescents who have substance use disorders are also more likely to drop out of school, and adolescents who drop out of school are also more likely to use substances [37]. Consistent with these issues, study participants overwhelmingly agreed on the need for educational and vocational assistance for AYA, suggesting that it should be a key component of AYA OUD treatment.

Results from this study can help create or improve treatment programs tailored to the developmental needs of AYA with OUD. Suggestions from study participants, combined with existing literature, indicate several considerations for developing OUD treatment programs for AYA. Results also point to the potential for modifying existing adult programs to better address AYA’s needs, which may be a more feasible interim step to address gaps in the availability of OUD treatment for this population than building new programs from the ground up. First, OUD treatment programming should be grouped by age to ensure the unique needs of AYA are addressed during treatment as well as facilitate peer connections during treatment and recovery. Treatment should also be comprehensive, including integration with primary care and access to social services such as housing and vocational development. Furthermore, the high prevalence of co-occurring mental health diagnoses among the AYA OUD population necessitates integrated substance use and mental health care. Providers need to receive more robust training to increase their knowledge and competency around treating OUD among AYA in a developmentally appropriate way that addresses their complex mental health concerns. Finally, AYA OUD treatment programs must create ample opportunities for family involvement across the continuum of care because family members play a critical role in shaping AYA behavior and supporting their treatment and recovery process [38].

In addition to the insights provided by key informants in this study, extant literature suggests a variety of complex individual, familial, and systemic factors, including systemic racism, that affect disparities in AYAs’ access to OUD treatment and caregiver involvement in treatment that need to be considered in future program development. For example, younger age, female sex, and non-White race are associated with both lower rates of receiving MOUD and higher rates of MOUD treatment dropout [39,40]. Furthermore, many youth who are on their caregivers’ health insurance plan may be unwilling to disclose their drug use to their family, and caregivers may be hesitant to consent for their child to receive MOUD [41], suggesting the need to navigate complex family dynamics in providing treatment to AYA. Regarding caregiver involvement in treatment, previous research has shown that caregivers can often feel overwhelmed by the issues their youths are going through, unsupported by the treatment program, or even feel blamed or judged by the youth’s providers [42]. Caregivers also cited logistical barriers to being involved in the youth’s treatment and a lack of knowledge about FBT [43].

This study has important limitations to consider. First, the study interviewed systems-level key informants, which did not include patients and their family members. Although key informants offer crucial systems-level feedback on the intricacies of the OUD treatment system, their perspectives cannot supplant lived experience. The study is thus an initial step toward program development, and future studies will directly collect feedback from AYA and their families. Second, it should be acknowledged that key informants were overwhelmingly White and female-identifying, which reflects the racial disparities of treatment providers in this area. Third, all key informants were from Rhode Island and Massachusetts, so results related to treatment availability and policy may not be generalizable to other regions. Fourth, due to the small number of OUD programs that serve youth younger than 18, and even fewer that focus only on adolescent OUD, not all key informants had direct experience with addressing OUD among minors.

## 5. Conclusions

Interview data from key informants of the OUD treatment system produced seven themes that reveal the unique treatment needs of AYA with OUD. Treatment programs that are developmentally tailored to AYA with OUD must include effective use of both MOUD and psychosocial treatments, opportunities for family involvement, as well as comprehensive wraparound services. AYA OUD treatment must also be highly integrated with other mental health treatments due to the high prevalence of co-occurring mental health diagnoses. Ensuring that AYA OUD treatment programs are tailored to the needs of this age group is key to increasing the availability of OUD treatment for a severely underserved AYA population. Ultimately, this study can contribute to treatment program development initiatives that will help decrease opioid-related morbidity and mortality among AYA with OUD.

## Figures and Tables

**Table 1:** Codes and corresponding semi-structured interview questions that were examined.

Code	Code Definition	Relevant Interview Question
2.1a Quality of available services for AYA	Comments and opinions about the adequacy or inadequacy of services available for AYA with OUD.	What treatment services are you aware of for AYA with OUD in the state?
2.2 Services needed for AYA and unmet treatment needs	Identified treatment needs for AYA with OUD that are not being addressed in the current system of care or services that we need more of. Can also include descriptions of programs that used to be offered and are no longer available.	What is your perception of the need for clinical services for AYA with OUD in this state?
4.1 Developmental needs and considerations for AYA	Comments about unique developmental considerations for AYA, including if current treatments are not a good fit for their needs.	What are some of the unique needs of AYA served by your organization?
5.1 Barriers and facilitators to providing treatment to AYA	What gets in the way of providing treatment to AYA as well as the strengths of the clinic that help them (or would help them) to treat AYA with OUD.	What barriers might there be to providing treatment to AYA with OUD in your organization?

**Table 2:** Demographics of study participants.

Variable	N	%
Age		
<25 years old	0	0.0%
25–34 years old	8	26.7%
35–44 years old	10	33.3%
45–54 years old	4	13.3%
55–64 years old	7	23.3%
65–74 years old	1	3.3%
75 years or older	0	0.0%
Gender Identity		
Male	6	20.0%
Female	24	80.0%
Non-binary/third gender	0	0.0%
Prefer not to say	0	0.0%
Hispanic/Latinx		
Yes	1	3.3%
No	29	96.7%
Race		
American Indian or Alaska Native	0	0.0%
Asian	0	0.0%
Black or African American	1	3.3%
White	28	93.3%
Native Hawaiian or Other Pacific Islander	0	0.0%
Other	2	6.7%
Years Practicing in Current Agency		
<1 year	4	13.3%
1–2 years	8	26.7%
>2–5 years	8	26.7%
>5–10 years	2	6.7%
>10–15 years	5	16.7%
>15 years	3	10.0%
Treats adolescents (<18 years)		
Treats adolescents	13	43.3%
Does not treat adolescents	17	56.7%
Primary Role		
Leader	10	33.3%
Provider	11	36.7%
Advocate	3	10.0%
State Policy	3	10.0%
Hospital Policy	3	10.0%
Level of Education		
Bachelor’s	10	33.3%
Master’s	11	36.7%
Ph.D.	6	20.0%
MD	3	10.0%

## Data Availability

The data presented in this study are available upon request from the corresponding author due to privacy.

## Abbreviations

The following abbreviations are used in this manuscript:

OUD	Opioid use disorder
AYA	Adolescents and young adults
MOUD	Medications for opioid use disorder
FBT	Family-based treatment
